# The thermal stress response of Aedes aegypti and Aedes albopictus when exposed to rapid temperature changes

**DOI:** 10.21203/rs.3.rs-6727380/v1

**Published:** 2025-05-26

**Authors:** Hunter Covey, Randall Wilson, Yaizeth Gurrola-Mares, Joseph R McMillan, Corey L. Brelsfoard

**Affiliations:** Texas Tech University; Texas Tech University; Texas Tech University; Texas Tech University; Texas Tech University

**Keywords:** population control, arboviruses, temperature, public health, thermal stress

## Abstract

Autocidal mosquito control approaches are based on rearing mosquitoes in consistent and stable laboratory conditions; however, when adult mosquitoes are released in field settings, they have to rapidly respond to changes in temperature and thermal stress. To examine the effect of thermal stress on mosquitoes, *Aedes aegypti* and *Aedes albopictus* larvae were reared at either 22°C (R22) or 28°C (R28) and emerged adults were subsequently exposed to temperatures of 22 (E22), 28 (E28), 32 (E32), or 38°C (E38). After the mosquitoes were subjected to these altered temperature profiles, we examined for effects on adult survivorship, respiration rates, and heat shock protein (HSP) expression. Reduced adult survivorship was observed when the rearing temperature was different than the adult exposure temperature. Respiration rates as a measure of thermal stress varied with rearing conditions and exposure temperatures, with notable declines observed as exposure temperatures increased. HSP gene expression was generally upregulated in response to thermal stress, with specific patterns differing by species and rearing conditions. Wing length measurements revealed no significant developmental differences across rearing temperatures, except for *Ae. aegypti* females. These findings highlight the impact of temperature on mosquito physiology and the potential impacts of rearing and environmental temperatures on the effectiveness of autocidal approaches for mosquito and disease control.

## Introduction

Autocidal mosquito control approaches have gained a significant amount of attention as effective novel forms of species-specific population and disease control for container inhabiting *Aedes* species [1–4]. Autocidal approaches include the incompatible insect technique (IIT), sterile insect technique (SIT), *Wolbachia*-based population replacement, release of insects carrying a dominant lethal genes (RIDL), and autodissemination augmented by adult male mosquitoes (ADAM) [5–8]. All aforementioned approaches rely on releasing males, females, or a combination of both sexes into variable, natural environments that can survive, find mates, and in some cases produce offspring. The dynamic nature of field settings is at odds with the mosquito manufacturing processes in which food amounts, humidity, rearing space, and temperature are optimized and held constant during larval development [9–11]. Studies show that exposure to rapid temperature shifts can lead to thermal stress, such that released mosquitoes may experience reduced activity, decreased survival rates, and alter vector competence due to thermal stress [12–14].

The impact of thermal stress on ectothermic invertebrates has been evaluated in several ways including the examination life history traits (e.g., longevity, survivorship, fecundity…etc.), measuring metabolic rate, and the upregulation of heat shock protein genes (Hsps), which can aid in the acclimation to environmental stressors and protect from oxidative stress and cellular damage [14–19]. In this study, we examined the effects of larval rearing temperatures and the effects of exposing adult *Aedes aegypti* (Linus) and *Aedes albopictus* (Skuse) to a different temperature than the larval rearing temperatures. Experiments were conducted with *Ae. aegypti* and *Ae. albopictus* to examine how rearing temperature and exposing adults to different temperatures impacted survivorship and longevity, respiration rates, and heat shock protein gene expression as a measure of thermal stress.

## Materials and Methods

### Mosquito rearing

*Aedes albopictus* used in the described experiments consisted of the NIAID, NIH strain from Gainesville, FL (MRA-804), contributed by Sandra A. Allan to BEI resources. *Aedes aegypti* used in these studies were from a colony strain collected from Corona, California. Eggs were hatched in 22 x 22 x 7 cm disposable plastic containers (Pactiv, Lake Forest, IL, USA) containing 500ml of aged liver powder solution (6g liver powder/L) (Now Foods, Bloomingdale, IL). Larval pans and adult cages were housed in an incubator set to 28 ± 2.2°C with a humidity of 80 ± 4% and a L:D cycle of 16:8 hours. After a 2 hr egg hatch interval, approximately 200 1st instar larvae were separated into rearing pans containing 500ml of DI water. Larvae were provided a 60g/L liver powder slurry for food ad libitum. Pupae were removed from larval rearing pans using a disposable plastic pipette and placed into 50 ml of DI water in a 140 mL plastic cups (Pactiv, Lake Forest, IL, USA). The plastic cup was then placed into a 24.5 x 24.5 x 24.5 cm BugDorm cage (MegaView Science Co., Taichung, Taiwan) where the pupae emerged into adults. Adults were provided with 10% sucrose solution ad libitum.

### Mosquito survivorship and longevity

To examine for an effect of thermal stress on adult survivorship and longevity, mosquitoes were reared at either 22 (R22) and 28°C (R28) and were exposed to either 22 (E22), 28 (E38) 32 (E32) and 38°C (E38) as adults. Seventy-five male and females mosquitoes were anesthetized using chloroform and placed in a 1.8 L mL plastic bucket modified to serve as mosquito cages (Airlite Plastics Co., Omaha NE). Each cage type consisted of 3–4 replicates. Due to the stress of anesthetizing the mosquitoes the mosquitoes were placed back in an incubator set to the initial rearing temperature for 24 hours. After the 24 hours, the cages were moved to the respective exposure temperature. The cages were monitored daily for dead adults.

### Mosquito respiration as a measure of thermal stress

For respiration experiments, *Ae. aegypti* and *Ae. albopictus* were reared as described in the previous section. Mosquito respiration rates were determined by a Li-Cor gas analyzer (LI-6800-89) with an attached insect chamber (Chamber volume 49.9cm3) (LICOR Corporate Offices - US, Lincoln, NE). Larvae were reared at either R22 or R28. Pupae were transferred to cages held at the same larval rearing temperatures and adults were allowed to emerge. Approximately 24 hours post emergence, 50 males or females were aspirated from their respective cage and placed into the Li-Cor respiration chamber. The Li-Cor gas analyzer pump speed was set to auto, the flow rate was set to 400μmol s-1, the press valve was set to 0.0 kPa, H2O was set to on, the relative humidity was set to 85%, CO2 injector was set to on, the soda lime was set to scrub auto, and the CO2_r was set to a setpoint of 400μmol-1. The adult mosquitoes were allowed to acclimate to the exposure temperature for 20 minutes before data collection began. A measurement of CO2 production was taken every one min for 120 min.

### Heat Shock Gene Expression

Real-time quantitative polymerase chain reaction (qPCR) was performed to examine the expression of heat shock genes: HSP26, HSP83 and HSC70. Primers for these genes are found in supplemental Supplementary Table 1 [20]. *Ae. aegypti* and *Ae. albopictus* whole mosquitoes from each rearing and exposure temperature combination at 2 and 24 hrs post exposure, were flash frozen using liquid nitrogen. RNA was extracted using Qiagen RNeasy kit following the manufacturer’s instructions. Following the RNA extraction, cDNA was made using a Lunascript cDNA synthesis kit (New England Biolabs, Ipswich, MA) following the manufacturer’s instructions. Thermocycler amplification cycling conditions consisted of an initial denaturing step of 95°C for 10 minutes followed by 40 cycles of, 95°C for 15 seconds, 54–60°C for 1 minutes,72°C for 30 seconds, and an elongation step at 72°C for 8 minutes.

### Wing Collection and Measurement

*Ae. albopictus* and *Ae. aegypti* were reared as described previously at R22 and R28. Once the mosquitoes reached adulthood, approximately 10–15 mosquitoes from both sexes were aspirated and placed into a 2.5mL Eppendorf tube and stored at −20°C in mineral oil to prevent desiccation (one for each sex and each rearing temperature). Wings were removed, placed on a slide, and imaged using an Leica S9 stereomicroscope and attached camera (Leica microsystems, Wetzlar, Germany). Wings were then measured using the images and ImageJ.

### Statistical analyses

Survivorship curves and log-rank analyses were performed using JMP Pro 17 (JMP Statistical Discovery LLC, Cary, NC). Comparisons of CO_2_ production from respiration trials was performed in R using a Generalized Estimation Equation model (GEE). Kruskal-Wallis tests followed by pairwise Wilcoxon tests were used to determine differences in the HSG gene expression and wing lengths. using JMP Pro 17 (JMP Statistical Discovery LLC, Cary, NC).

## Results

### The Effects of thermal stress on adult survivorship and longevity

**Figure 1:**
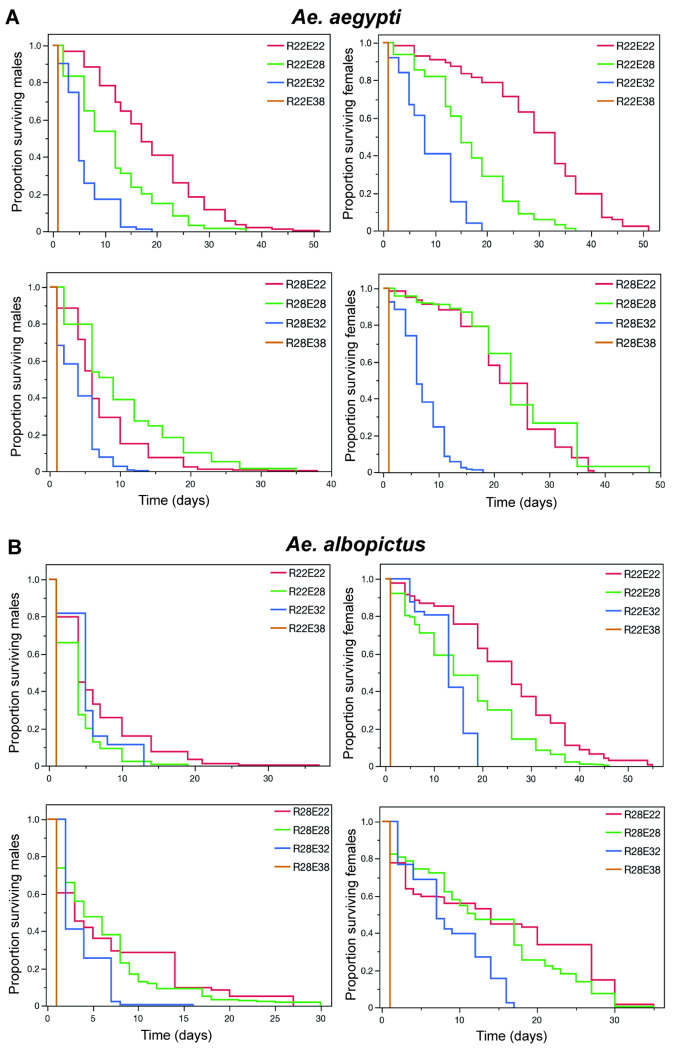
*Ae. aegypti* and *Ae. albopictus* mean survivorship after reared at R22 and R28°C and exposed to 22°C, E28, E32 and E38°C. Each rearing and exposure temperature combination is represented by a different color line in each plot.

To examine for an effect of rearing temperature on adult survivorship at different exposure temperatures, *Ae. aegypti* females and males were reared at R22 and R28 and exposed as adults to the following temperatures, E22, E28, E32 and E38. The survivorship of both *Ae. aegypti* and *Ae. albopictus* males and females was generally reduced when the rearing temperature was different than the exposure temperature (Bonferroni corrected Log-Rank tests, P < 0.008) (Fig. 1 and Supplementary Table 2). It is interesting to note that at E38, that *Ae. aegypti* and *Ae. albopictus* females and males did not survive longer than 24-hrs post exposure to 38°C (Fig. 1). To determine if the survivorship analysis was influenced by high early mortality rate in the 38°C exposure treatments, the overall log rank tests were repeated within groups without the 38°C data and results were similar (Bonferroni corrected Log-Rank tests, P < 0.001).

### The Effects of thermal stress on mosquito respiration

**Figure 2:**
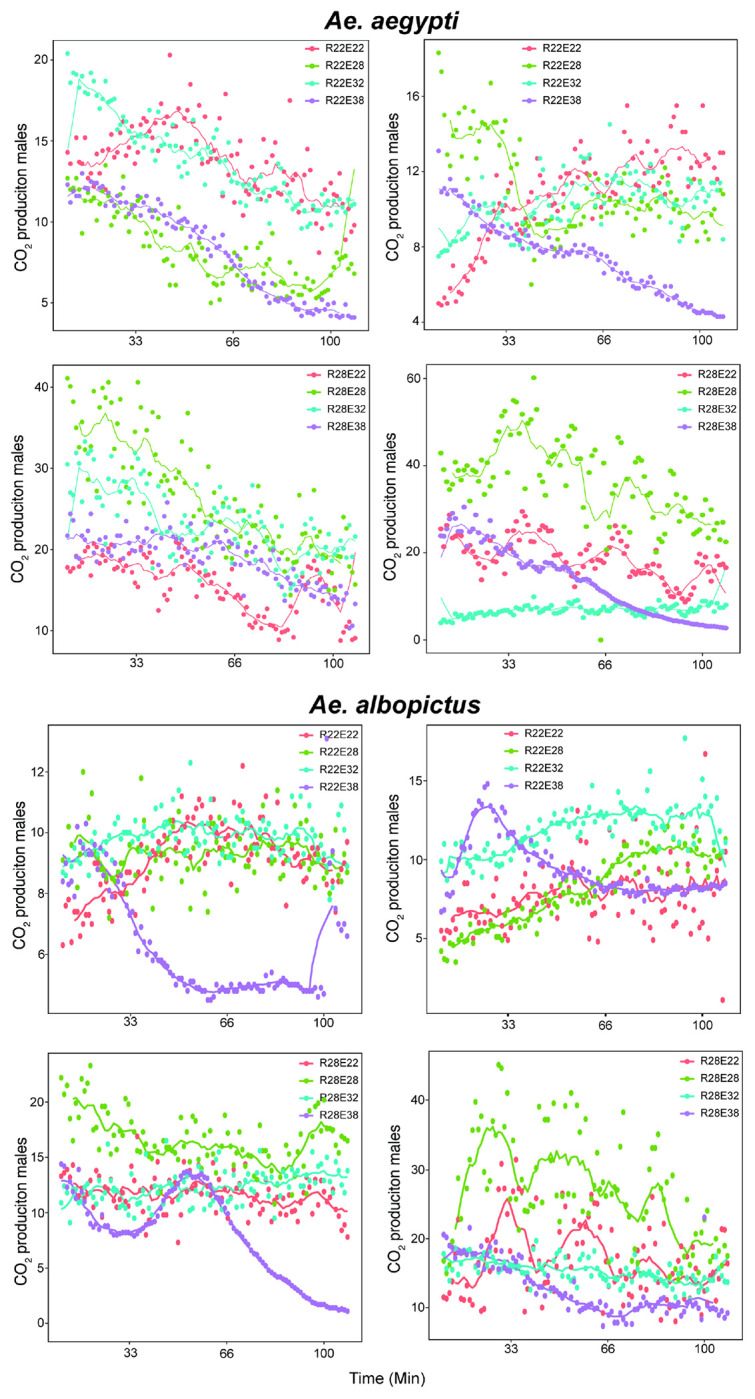
Adult respiration was used as a proxy for mosquito stress when exposed to different temperatures. CO2 production was measured for each rearing and exposure temperature combination for male and female (A) *Ae. aegypti* and (B) *Ae. albopictus* over a 120 min time period.

When *Ae. aegypti* and *Ae. albopictus* males and females were exposed to a different temperature than what they were reared at there was a measurable reduction in respiration rates, (GEE, P < 0.0001) (Supplementary Table 3), which suggests the mosquitoes are experiencing thermal stress associated with exposure to temperature changes (Fig. 2). To determine if the observed differences in respiration rate was influenced by potential mortality in the 38°C exposure treatments, the overall log rank tests were repeated within groups without the 38°C data and results were similar (GEE, P < 0.001).

### Expression of Heat Shock Genes as a result of thermal stress

**Figure 3:**
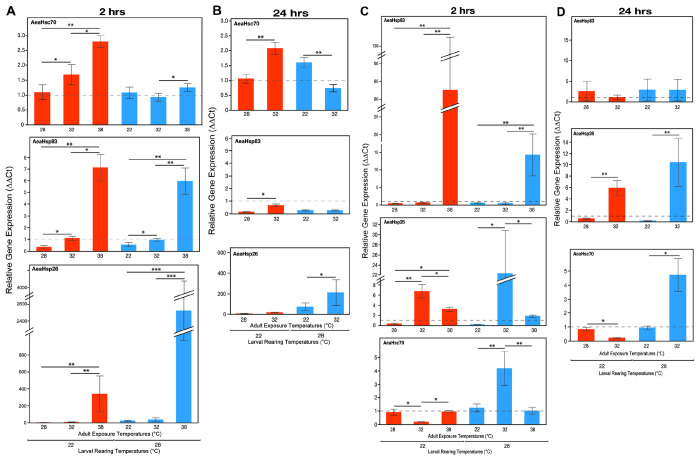
HSG expression levels of *Aedes aegypti*(A) 2 hrs and (B) 24 hrs and *Ae. albopictus* (C) 2 hrs and (D) 24 hrs post exposure to temperatures different than their rearing temperature. Lines with asterisks above the bars significant differences according to Bonferroni corrected pair-wise comparisons within each rearing temperature group (* P ≤ 0.01, ** P ≤0.001, *** P ≤ 0.0001).

When *Ae. aegypti* and *Ae. albopictus* mosquitoes were exposed to a different temperature than their rearing temperature, particularly at a temperature extreme of 38°C a collective increase in the expression of AeaHSP83, AeHsp70, and AeaHsp26 was observed (Kruskal-Wallis, P < 0.05) (Fig. 3). All comparisons with each rearing temperature and post adult exposure times are shown in Supplementary Table 4. Overall, the changes in expression levels were more pronounced at 2 hrs post exposure rather than 24 hrs post exposure.

### Effect rearing temperature on adult size

**Figure 4:**
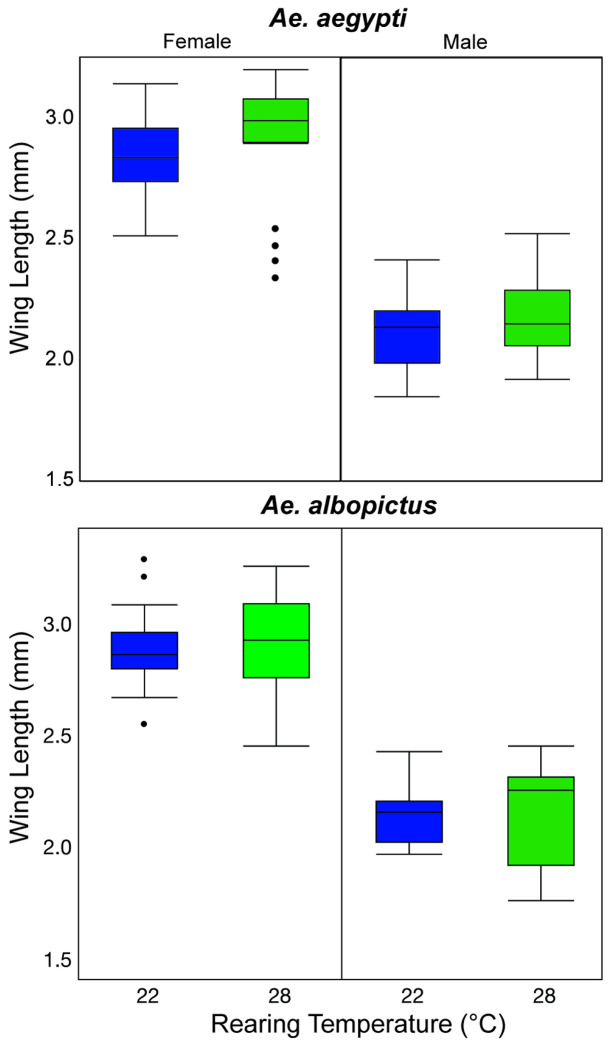
Wing length measurements for *Ae. aegypti* and *Ae. albopictus* males and females reared at 22 or 28 °C. No significant difference was observed when comparing the wing length of each species and sex (P < 0.05). The upper and lower quartile values are the top and bottom of each box and the median is represented by the line in each box. Error bars are standard deviation.

When comparing the two different rearing temperatures, there was no observed impact on the adult size of *Ae. aegypti* (Kruskal-Wallis, ChiSquare = 0.77, DF = 1, P = 0.38) or *Ae. albopictus* (Kruskal-Wallis, ChiSquare = 0.41, DF = 1, P = 0.52) (Fig. 4).

## Discussion

Our findings demonstrate that thermal stress significantly impacts the survivorship and longevity of *Ae. aegypti* and *Ae. albopictus* adults, with reduced survival observed when there is a mismatch between rearing and exposure temperatures. Both species exhibited optimal survivorship when rearing and exposure temperatures were the same (i.e., R22–E22 and R28–E28), suggesting a degree of thermal acclimation that enhances thermal tolerance. Conversely, exposure to temperatures that differed from rearing conditions, particularly at extreme temperatures such as 38°C, dramatically reduced survival, with no individuals surviving beyond 24 hours post-exposure. This aligns with previous studies indicating that exposure to high thermal extremes can exceed physiological limits, resulting in rapid mortality [14].

Interestingly, when using respiration as a proxy to measure thermal stress, we observed a unexpected reduction in respiration rates when mosquitoes reared at one temperature and later exposed to either lower or higher temperatures as adults. While an increase in temperature was hypothesized to correlate with higher respiration rates, *Ae. aegypti* and *Ae. albopictus* males and females exhibited lower respiration rates when exposed to temperatures different from their rearing conditions. The pattern of CO_2_ release in mosquitoes reflects their metabolic state, with discontinuous gas exchange (DGE) occurring during rest phases. When activity levels increase, DGE transitions to continuous gas exchange, reflecting heightened metabolic demands. This suggests that under thermal stress, mosquitoes may adopt a strategy of reduced metabolic activity, potentially seeking out resting sites to conserve energy until conditions become more favorable. These findings align with previous studies, which reported that mosquitoes prefer microhabitat resting areas that are cool, have higher humidity, and where temperature variation is minimal before becoming active [21, 22]. Our respiration data further support the hypothesis that temperature shifts influence mosquito metabolism. When mosquitoes were maintained at the same temperature from larval to adult stages, respiration rates were higher compared to those experiencing a temperature shift. This suggests that any deviation from the rearing temperature imposes metabolic stress, leading to reduced activity and energy conservation [23, 24]. Exposure to higher temperatures in adulthood compared to larval conditions resulted in a dormancy-like response, lowering respiration rates and minimizing energy expenditure. While the E38 treatment had the most pronounced reduction in respiration rates, the observed dormancy-like response was not driven only by this temperature extreme but was also evident in each temperature shift from larval to adult exposure temperature.

As an additional measure of thermal stress the expression of HSG was also examined. When comparing the expression of HSG relative to the treatment with the same rearing and adult exposure temperature there was an increase in HSG expression when there was a temperature shift higher than the rearing temperature. Consistently, the E38 treatment induced upregulation of heat shock genes across both species, indicating a thermal stress response associated with exposure to a detrimental temperature. The increase of expression of HSG associated with an increase in exposure temperature suggests that mosquitoes activate protective mechanisms to counteract thermal stress. Previous research has shown that *Ae. aegypti* and *Ae. albopictus* larvae exposed to high temperatures (e.g., 37°C and 39°C) developed increased thermotolerance upon re-exposure to even higher temperatures (43°C and 45°C) [18, 25]. This suggests that heat shock proteins (HSPs) play a crucial role in buffering the effects of thermal stress.

Additionally, we investigated whether larval rearing temperature influenced adult size by measuring wing length. The data showed that significant differences in wing length were only observed in *Ae. aegypti* females, while other groups exhibited no notable variation. This suggests that rearing temperatures of 22°C and 28°C had no impact on adult mosquito size, and that rearing at 22 or 28 °C ultimately did not result in any subsequent effects when adults were exposed to the different temperature regime.

Here we have demonstrated that rapid temperature shifts mosquitoes may experience while being released as part of an autodissemination approach may temporarily impact the initial success of the releases depending upon the autocidal approach being utilized. These results suggest that the temperature at the time of mosquito release is a crucial factor to consider for autocidal approaches. Although ambient temperatures cannot be controlled, release timing can be optimized to improve dispersal and survivorship. To mitigate thermal stress, releases should ideally occur during the early morning or late evening in temperate and tropical regions where release temperatures are similar to their laboratory or factory rearing temperatures. Furthermore, optimizing rearing and pre-release temperatures to match ambient conditions could enhance the fitness and effectiveness of released individuals. Evidence suggests that environmental conditions experienced at one life stage influence subsequent stages, emphasizing the importance of temperature adaptation across developmental phases [26]. Our experiments utilized laboratory-reared strains maintained for over 30 generations, raising the question of whether mosquitoes from natural populations with different genetic backgrounds might exhibit varying responses to temperature stress. Future studies should investigate the thermal adaptability of field-collected mosquitoes from diverse climatic regions to assess potential local adaptations. Moreover, the respiration experiments were unreplicated, and future studies could include additional replicates of strains with different genetic backgrounds.

Overall, our finding are similar to other previous studies and suggest that *Ae. aegypti* and *Ae. albopictus* have a narrow thermotolerance range and rely on genetic and behavioral responses to mitigate thermal stress [12, 16, 20, 25, 27, 28]. Understanding these adaptive strategies can improve the development and release of more robust mosquitoes for autocidal control methods.

## Abbreviations

R22: reared at 22 °C
R28: reared at 28 °C
E22: exposed to 22 °C
E28: exposed to 28 °C
E32: exposed to 32 °C
E38: exposed to 38 °C
HSP: heat shock protein
SIT: sterile insect technique
RIDL: release of insects carrying a dominant lethal genes
ADAM: autodissemination augmented by male mosquitoes
qPCR: Real-time quantitative polymerase chain reaction
DGE: discontinuous gas exchange
